# The dual role of miR-184 in cancer: a systematic review of context-dependent regulation

**DOI:** 10.1007/s11033-025-11314-4

**Published:** 2025-12-11

**Authors:** Brandon Duff, Rasha Swadi

**Affiliations:** 1 School of Pharmacy and Biomolecular Sciences, Liverpool John Moores University, Liverpool, UK; 2 School of Biological Sciences, University of Reading, Reading, UK; 3 Institute for Health Research, Liverpool John Moores University, Liverpool, UK

**Keywords:** MicroRNA-184 (miR-184), Context-dependent regulation, Therapeutic biomarker, LncRNA–ceRNA network, Tumour suppressor

## Abstract

MicroRNA-184 (miR-184) regulates gene expression by binding to target messenger RNAs, promoting their degradation, and influencing key cellular processes. Its role in human cancers is complex: while several studies report tumour-suppressive effects, including inhibition of proliferation, migration, and metastasis, contradictory evidence indicates that miR-184 may act as a tumour promoter in certain contexts. This systematic review aimed to clarify whether miR-184 functions as a universal anticancer agent across human cancers. A comprehensive search of PubMed and SCOPUS identified 123 records, of which 55 studies met inclusion criteria. Analysis revealed that miR-184 predominantly acts as a tumour suppressor in cancers such as prostate and breast, whereas in liver and bone cancers, it exhibits tumour-promoting activity. Certain cancer types, including skin and pancreatic cancers, showed inconsistent or context-dependent effects. Key molecular targets and pathways influenced by miR-184, including c-MYC, caspases, and apoptotic signalling, were highlighted. Overall, these findings demonstrate that the function of miR-184 in cancer is context-dependent, shaped by tissue type, molecular environment, and cellular signalling networks.

## Introduction

MicroRNAs (miRNAs) are small, non-coding RNA molecules, approximately 22 nucleotides in length, that regulate gene expression post-transcriptionally. They typically bind complementarily to the 3′ untranslated region (3′UTR) of target messenger RNAs (mRNAs), leading to mRNA degradation or translational inhibition [1]. Through this regulatory mechanism, miRNAs play crucial roles in diverse cellular processes, including cell cycle regulation, apoptosis, cell migration, and angiogenesis [2]. These processes are critical in cancer development and progression, making miRNAs an important focus in cancer biology, prognosis, and therapeutic research.

**Fig. 1:**
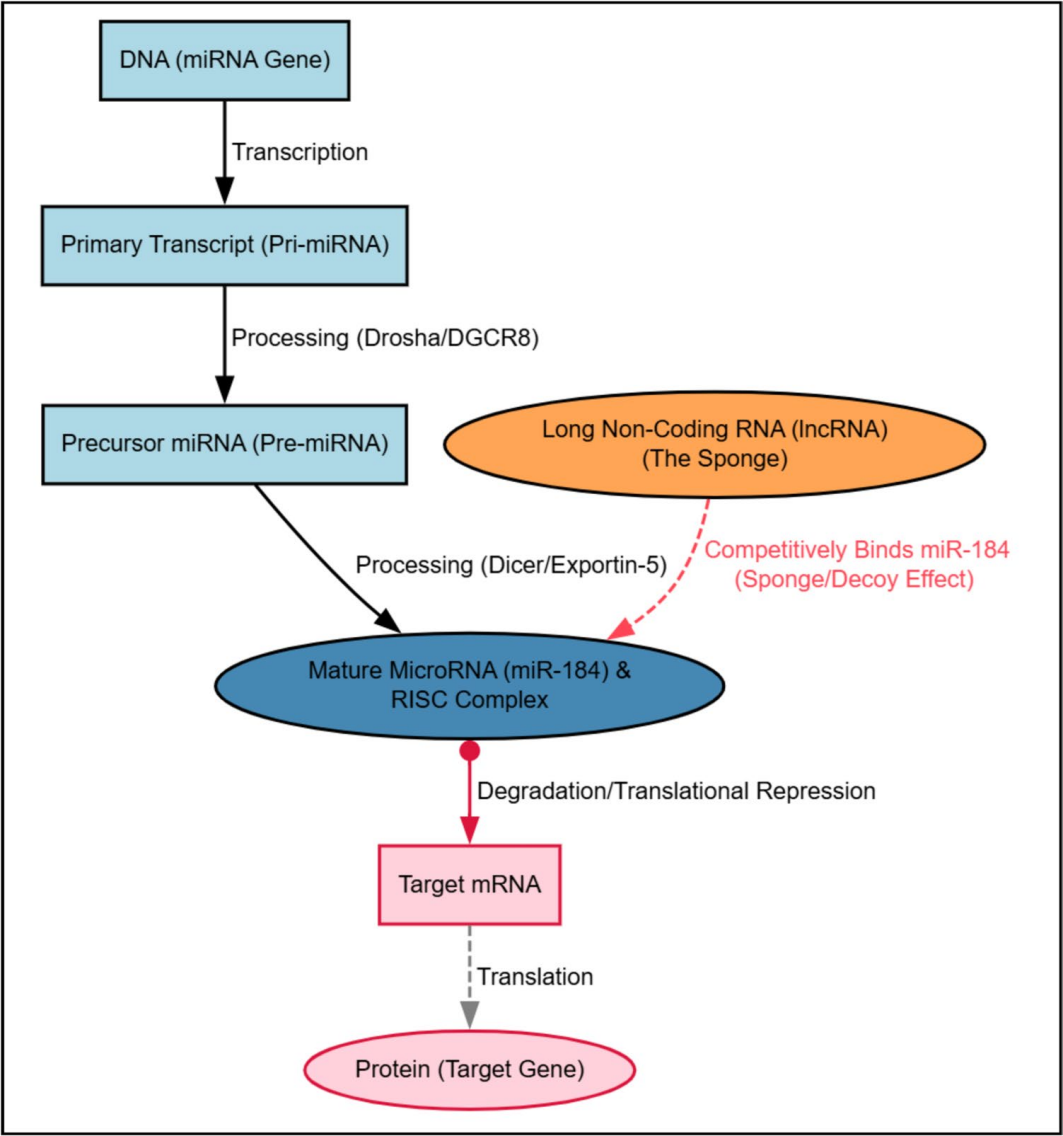
MiR-184 biogenesis and lncRNA regulatory network. the figure illustrates the multi-step biogenesis of mature microRNA-184 (miR-184). transcription of the DNA gene produces a primary transcript (Pri-miRNA), which is processed by the microprocessor complex (drosha/DGCR8) into a precursor hairpin (Pre-miRNA). this Pre-miRNA is exported to the cytoplasm, where dicer excises the mature miR-184 duplex. the mature miR-184 is incorporated into the RISC complex to target mRNA for degradation or translational repression. crucially, the diagram highlights the competitive endogenous RNA (ceRNA) mechanism: long non-coding RNAs (lncRNAs) act as ‘sponges’ by competitively binding and sequestering mature miR-184, thereby reducing the amount of miR-184 available to inhibit the target mRNA. This ceRNA sequestration is a key mechanism driving the context-dependent functional switch of miR-184 in cancer

The production of mature miRNAs, known as biogenesis, is a highly regulated multi-step process involving the Microprocessor complex (Drosha/DGCR8) in the nucleus and the Dicer enzyme in the cytoplasm (Fig. 1). Crucially, this regulatory pathway can be modulated by non-coding RNAs, particularly long non-coding RNAs (lncRNAs). These lncRNAs can act as competitive endogenous RNAs (ceRNAs), or ‘sponges’, by binding and sequestering miRNAs [3]. This ceRNA mechanism is vital, as it effectively reduces the amount of free miRNA available to target mRNAs, serving as a powerful mechanism to switch a miRNA’s functional output from a tumour suppressor to a tumour promoter in a context-dependent manner.

Among these molecules, microRNA-184 (miR-184) functions by regulating gene expression through degradation or translational inhibition of target mRNAs [4]. In normal human tissues, miR-184 is primarily expressed in the eye and central nervous system, where it contributes to the development and differentiation of ocular and neural tissues [5, 6].

Increasing evidence indicates that miR-184 exerts tumour-suppressive effects across several malignancies, including skin [7], breast [8], prostate [9], and lung cancers [10]. This tumour suppression is often mediated by promoting apoptosis and preventing uncontrolled cell proliferation, one of the hallmarks of cancer [11]. Furthermore, miR-184 inhibits cell invasion and metastasis by regulating the epithelial-to-mesenchymal transition (EMT). EMT enables epithelial cells to lose adhesion and acquire migratory capabilities. miR-184 upregulates E-cadherin, a key cell–cell adhesion molecule that suppresses EMT, thereby reducing tumour invasion, migration, and metastasis [12].

Experimental evidence suggests that miR-184 exerts its tumour-suppressive functions by targeting oncogenes, modulating tumour suppressors, inhibiting metastasis, and suppressing angiogenesis, both in vitro and in vivo [13]. For instance, Zhu et al. [12] demonstrated that miR-184 inhibits the invasive potential of nasopharyngeal carcinoma (NPC) cells by suppressing Notch2-mediated EMT. Similarly, Phua et al. [14] reported that miR-184 acts as a tumour suppressor in breast cancer (BC), reducing primary tumour initiation through the inhibition of cell division, evidenced by decreased proliferation markers and mitotic figures. This antiproliferative effect was also observed in triple-negative breast cancer (TNBC) models.

miR-184 has emerged as a potential therapeutic target across a range of malignancies, from lymphomas to carcinomas. However, the magnitude and consistency of its antitumour effects vary among cancer types. For example, the tumour suppressor p53, a central regulator of apoptosis, is inhibited by the inhibitory member of the apoptosis-stimulating protein of p53 (iASPP), which is upregulated in central nervous system lymphoma (CNSL). miR-184 suppresses iASPP by binding to its 3′UTR, resulting in decreased cell survival, invasion, and tumour volume via modulation of the phosphatidylinositol 3-kinase (PI3K)/AKT signalling pathway [15]. Similarly, studies in renal cell carcinoma (RCC) have shown that miR-184 reduces proliferation and migration while inducing apoptosis of malignant cells, suggesting therapeutic potential in treatment-resistant cancers such as advanced RCC [16].

Despite substantial evidence supporting its tumour-suppressive role, miR-184 exhibits context-dependent functions, acting as an oncogene in certain malignancies [17–19]. For example, Wu et al. [22] found that miR-184 promotes hepatocellular carcinoma (HCC) progression by enhancing tumorigenicity, proliferation, and cell cycle progression through modulation of the Wnt/β-catenin signalling pathway. Likewise, in osteosarcoma, miR-184 has been shown to enhance metastatic, invasive, and proliferative capabilities in both in vitro and in vivo models [19]. These contradictory findings underscore the complexity of miR-184’s role in cancer biology and the necessity for systematic evaluation.

Given this landscape of conflicting evidence, a simple narrative review is insufficient to resolve the functional discrepancy of miR-184. Therefore, this systematic review goes beyond previous summaries by focusing on three critical objectives: (1) Direct reconciliation of contradictory evidence, where we synthesise opposing functional roles across specific cancer contexts to establish a comprehensive, context-dependent functional profile; (2) Critical quality assessment, involving an objective appraisal of the methodological quality of all included studies to ensure high-confidence evidence is prioritised; and (3) Translational consistency assessment, where we compare findings between in vitro and in vivo models to determine the robustness and therapeutic viability of miR-184 targeting. These focused objectives address the lack of critical synthesis and methodological rigour in previous publications, providing a clear foundation for future research and clinical exploration.This systematic review aims to comprehensively examine and clarify the role of miR-184 across various cancer types and experimental models. Specifically, it seeks to reconcile discrepancies in existing literature and identify high-quality evidence addressing the central research question: *“****Does*** ***miR-184*** ***act*** ***as*** ***an*** ***antitumour*** ***agent*** ***across*** ***all*** ***human*** ***cancers?”***

The key objectives of this study are to:

I. Examine evidence supporting miR-184’s tumour-suppressive effects across different human cancers.
II. Identify signalling pathways and cellular processes regulated by miR-184 in tumour suppression.
III. Assess the quality and consistency of existing evidence across experimental models (in vitro, in vivo* and computational*).
IV. Evaluate the context-dependent functions of miR-184 in tumour promotion versus suppression.
V. Determine the therapeutic potential of miR-184 as a target for novel cancer treatments.

Understanding the dualistic role of miR-184 in cancer biology has major clinical implications for diagnosis, prognosis, and therapy development. With approximately ten million cancer-related deaths occurring worldwide each year, cancer remains one of the greatest challenges to global health in the twenty-first century [23]. The pursuit of effective novel therapeutic strategies is therefore critical for improving clinical outcomes. This review aims to bridge existing knowledge gaps, resolve current inconsistencies in the literature, and provide a comprehensive synthesis of miR-184’s complex role in cancer, thereby guiding future translational and clinical research efforts.

## Methodology

### Research questions

#### Primary research question: does miR-184 exhibit an antitumour role across all human cancers?

##### Sub-questions

I. In which types of cancers does miR-184 most effectively act as a tumour suppressor?
II. Which cellular processes are influenced by miR-184 to induce tumour suppression (e.g., cell cycle regulation, apoptosis, inhibition of tumour invasion and metastasis, inhibition of angiogenesis, or modulation of tumour suppressor genes and oncogenes)?
III. Which target molecules and signalling pathways are regulated by miR-184 to mediate its antitumour effects?
IV. What are the direct molecular targets of miR-184, and can they be manipulated for therapeutic benefit in cancer treatment?

### Search strategy

#### Search query

The following Boolean search string was used to retrieve relevant studies:

(“miR-184” OR “microRNA-184” OR “miRNA-184”) AND (“anticancer” OR “antitumour” OR “antitumor” OR “tumour suppression” OR “tumor suppression” OR “tumour suppressor” OR “tumor suppressor”).

### Search terms

- “miR-184” is the standard nomenclature for this miRNA, while “microRNA-184” and “miRNA-184” are widely used synonyms in scientific literature. Including all variants ensured comprehensive coverage of relevant publications.
- “Anticancer” and its variants (“antitumour” and “antitumor”) are frequently used interchangeably across journals. Both British and American spellings were included to ensure capture of studies from different regions.
- “Tumour suppression” (or “tumor suppression”) describes the inhibition of cancer cell growth and proliferation. Similarly, “tumour suppressor” (or “tumor suppressor”) refers to genes or molecules that prevent cancer development. Including both terms allowed identification of studies specifically investigating miR-184’s tumour-suppressive role.

These terms were selected to capture studies examining both the therapeutic and preventive anticancer functions of miR-184.

### Boolean operators

- **OR**: Used to connect synonymous or related terms, ensuring that all studies using different terminologies were included.
- **AND**: Used to combine essential components of the query, ensuring retrieved studies specifically investigated miR-184 in relation to anticancer or tumour-suppressive functions.

This structured Boolean approach maximised sensitivity while maintaining relevance to the primary research question.

### Inclusion and exclusion criteria

#### Inclusion criteria

- **Publication Date:** Studies published between *2014 and 2024* to ensure inclusion of the most current and relevant research.
- **Study Type:** Original experimental studies providing empirical data; review articles and meta-analyses were excluded.
- **Search Term Relevance:** Articles had to include the specified keywords, *(miR-184 OR microRNA-184 OR miRNA-184)* AND *(anticancer OR antitumour OR antitumor OR tumour suppression OR tumor suppression OR tumour suppressor OR tumor suppressor)*, to ensure direct relevance.
- **Language:** English-language publications only, due to language proficiency and consistency in data interpretation.

### Exclusion criteria

- Publications outside the specified date range.
- Review articles, conference abstracts, editorials, and commentaries.
- Non-English language papers.
- Studies lacking experimental evidence directly related to miR-184 and cancer.

### Data extraction and quality assessment

This systematic review was conducted according to the PRISMA (Preferred Reporting Items for Systematic Reviews and Meta-Analyses) 2020 guidelines [24]. The PRISMA framework was applied to ensure methodological transparency and reproducibility.

A structured process was followed to identify, screen, and include eligible studies:

1. **Identification:** Databases were searched using the predefined Boolean search string. Duplicates were identified and removed.
2. **Screening:** Titles and abstracts were independently reviewed to determine relevance based on inclusion criteria.
3. **Eligibility:** Full-text articles of potentially relevant studies were retrieved and assessed for eligibility.
4. **Inclusion:** Studies meeting all inclusion criteria were included in the final qualitative synthesis.

**Fig. 2:**
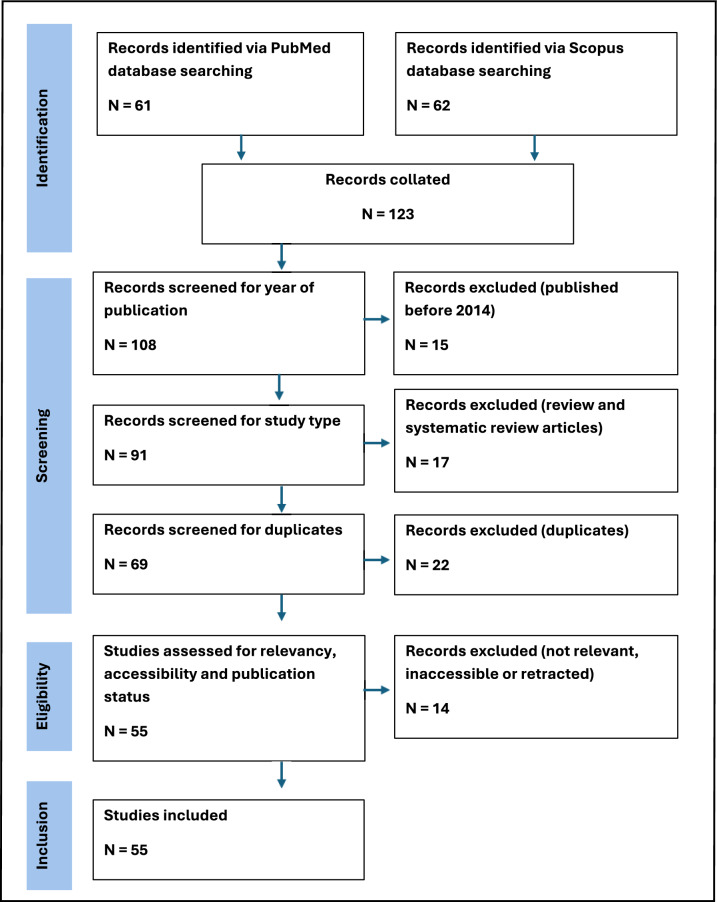
PRISMA 2020 flow diagram illustrating the study selection process. the diagram shows the number of records identified through database searches in PubMed and Scopus (n = 123), the sequential screening and exclusion steps, and the final number of studies included in the systematic review (n = 55)

A PRISMA flow diagram (Fig. 2) summarises the number of studies identified, screened, assessed for eligibility, and included in the review.

### Data extraction

Data were systematically extracted from each eligible study and recorded in a structured table. Extracted information included:

- Author(s), year of publication, and country
- Cancer type and model (in vitro, in vivo, or patient-derived/clinical)
- Experimental methods and key findings
- Target genes and signalling pathways associated with miR-184
- Reported functional effects (e.g., proliferation, apoptosis, migration, invasion, angiogenesis)

### Quality assessment

Each included study was evaluated for methodological quality and risk of bias based on PRISMA recommendations and standardised assessment criteria, focusing on:

- Clarity of research objectives
- Appropriateness of experimental design
- Validation of molecular assays
- Statistical analysis robustness
- Reproducibility of findings

Studies were categorised as high, moderate, or low quality based on the completeness and reliability of their methodological reporting. Discrepancies between reviewers were resolved through discussion until consensus was reached.

### Data tabulation and visualisation

Data were synthesised and visualised for clarity and comparison across studies.

- **Figures:** Figs. 1, 2 and 5 were created using *Microsoft PowerPoint*; Fig. 6 was generated using *Canva*; other figures were produced in *Microsoft Excel*.
- **Tables:** Table 1 was prepared using *Microsoft Word* for consistency in layout and readability.

**Table 1:** Summary of the characteristics of the 55 included studies. details include cancer type, overall effect of miR-184 on tumours (suppression (-), promotion (+), or undetermined), experimental models used, investigation methods, main outcomes, and clinical potential (if reported)

Cancer type	Promotion or suppression (+ or -)	Experimental models	Investigation methods	Significance/main outcomes	Clinical potential	References
Lung	-	72 SCLC tissue samples Cell lines	Microarray analysis, qRT-PCR, 3’UTR reporter assay, immunoblotting, functional assays on Cell Lines	miR-184 suppressed tumour invasion and metastasis via β-catenin by targeting EPAS1	Therapeutic	[25]
	-	Cell lines 124 NSCLC tissue samples	PCR and site-directed mutagenesis, boyden chamber assay, RT-PCR analysis, kaplan–meier and Cox regression analysis	miR-184 acts as a tumour suppressor by targeting CDC25A and c-MYC, inhibiting cell proliferation and invasion	Prognostic and therapeutic	[26]
	-	136 NSCLC tissue samples Cell lines	miRNA manipulation and transfection, RT-PCR, luciferase reporter assay, ChIP assay, MTT cytotoxicity assay	miR-184 suppression by E6 oncoprotein confers cisplatin resistance by increased Bcl-2 expression	Prognostic and therapeutic	[27]
	-	108 lung cancer tissue samples Cell lines	RNA isolation, RNA Library creation and Sequencing, miRNA validation, target identification, functional studies on cell lines	miR-184 was significantly downregulated in both SCC and AC tissues compared to normal lung tissues	Unspecified	[28]
	-	Cell lines 73 NSCLC and 22 non-cancerous tissue samples Nude mice	Cell culture, western blot & IHC, lentivirus & transfection, RNA analysis, cell assays, in vivo experiments, miRNA & protein analysis, statistical analysis	miR-184 acts as a tumour suppressor in NSCLC. It suppresses cell proliferation by targeting c-MYC and modulating cell cycle regulators	Therapeutic prognostic	[10]
	-	Cell lines 24 nude mice	Protein analysis, viability assays, animal model experiments, gene targeting	miR-184 suppressed EMT	Therapeutic	[29]
	Undetermined	Computational approach	Data collection, miRNA analysis, gene prediction and mutation analysis, pathway and diagnostic analysis, correlation analysis	miR-184 demonstrated potential for early LUAD diagnosis	Prognostic	[30]
	Undetermined	Computational approach	miRNA array analysis, target prediction, signalling pathway mapping, functional classification, survival analysis	Multiple miRNAs were identified as inhibiting EGFR-targeted drugs, including miR-184	Prognostic	[31]
Liver	+	Cell Lines 10 NSG mice	RNase R treatment, qRT-PCR, Cell proliferation, migration, invasion, apoptosis, and dual-luciferase reporter assays, in vivo experiments	circITCH functions as a molecular sponge for miR-184, which is upregulated in HCC. This inhibits miR-184’s oncogenic effects	Therapeutic	[32]
	+	Cell lines 136 tissue samples 10 BALB/c nude mice	CCK8, colony formation, EDU incorporation, flow cytometry, transwell, and scratch wound healing assays, tumour mouse xenograft assays, dual-luciferase reporter, RNA pull-down, RNA immunoprecipitation, and western blotting assays	circ-102,166 suppresses HCC progression by sponging miR-182 and miR-184	Therapeutic	[33]
	+	Cell lines 10 HCC tissues 10 adjacent healthy tissues 15 male athymic BALB/c nude mice	RNA isolation, RNA and protein analysis, qRT-PCR transfection, cell proliferation assays, migrations assays, cell cycle and apoptosis analysis	LINC00205 acted as a ceRNA sponging miR-184, upregulating EPHX1 expression. overexpression of EPHX1 or inhibition of miR-184 reduced tumorigenicity and cell proliferation	Therapeutic	[34]
	-	Cell lines 20 BALB/c nude mice	miRNA target prediction – online programmes, network analysis, luciferase reporter assays, qRT-PCR, western blot, cell proliferation (MTT), invasion (transwell), migration (wound healing) assays	miR-184 targets AKT2, a key player in the AKT/mTOR pathway miR-184, and other miRNAs, regulate pathways in HCC progression, such as cell cycle, apoptosis, and mTOR signalling Validated targets of miR-184 were enriched in oncogenic pathways. This implied miR-184 acted as a tumour suppressor in HCC	Therapeutic	[35]
	Undetermined	Computational approach	miRNA target prediction, Validation, Pathway enrichment analysis	miR-184 may play a role in the regulation of RASGRF1, could be relevant to the pathological mechanisms underlying HCC	None	[36]
	Undetermined	Cell lines THP-1 monocytes and HCC cells 169 HCC and adjacent tissue samples 8 nude mice	Exosome isolation, high-throughput sequencing, in vitro experiments, bioinformatics analysis, qPCR, western blotting, flow cytometry	miR-184 was the most upregulated miRNA in exosomes from HCC cells treated with tunicamycin Not associated with PD-L1 expression Increased expression of ER stress markers negatively correlated with overall survival and clinicopathological scores	None	[37]
	+	Cell lines tissue samples: 8 pairs of HCC and adjacent	Transfection, RNA Isolation, qRT-PCR, western blotting, luciferase assay, MTT assay, colony formation assay, anchorage-independent growth ability assay, flow cytometry, statistical analysis	Ectopic expression of miR-184 enhances cell proliferation, tumorigenicity, and cell cycle progression in HCC cells Inhibition of miR-184 reduces these effects, indicating its role in promoting HCC progression	Therapeutic	[22]
Brain	-	Cell lines Glioma tissue samples Athymic nude mice	Bioinformatics, Sample analysis, transfection, in vivo experiments, PCR, western blotting, luciferase assays, Functional assays	Overexpression of miR-184 in glioma cells inhibited invasion, colony formation, and anchorage-independent growth, indicating its tumour-suppressive role	Therapeutic	[38]
	-	Cell lines 12 nude mice	Transfection, RNA extraction, functional assays, protein analysis, RNA analysis, in vivo experiments	miR-184 inhibited cell proliferation and invasion, induced apoptosis and caused cell cycle arrest In vivo tumours with overexpressed miR-184 were smaller	Therapeutic	[39]
	-	Cell Lines: glioma and BC	Transfection, MTS assay, invasion and adhesion assays, cell cycle assay, real-time PCR, western blotting, ELISA, report gene assay, statistical analysis	miR-184 suppressed proliferation and invasion of human glioma and BC cells and induced cell cycle arrest	Therapeutic	[40]
	-	Tissue samples: 76 astrocytoma specimens (50 GBMs, 13 AAs, 13 DAs) and 10 non-neoplastic brain tissues Cell lines	Cell culture, RNA extraction and analysis, statistical analysis	Low miR-184 associated with aggressive clinicopathological features, such as advanced tumour grade, older patient age, lower Karnofsky performance score, and higher Ki-67 index	Prognostic	[41]
	+	Cell Lines	Transfection, western blotting, MTT assay, hoechst staining, RT-PCR, luciferase assay, apoptosis assay, caspase activity, invasion assay, migration test, statistical analysis	Overexpression of miR-184 enhances cell viability, inhibition of miR-184 reduces cell viability and induces apoptosis miR-184 upregulation promotes malignant behaviour of glioma cells, including increased invasion and migration capabilities	Therapeutic	[42]
	Undetermined	73 Pituitary adenoma tissue samples (13 GHPAs, 42 NFPAs, and 18 PRLPAs) and 6 normal pituitary glands	RNA extraction, sequencing, annotation, validation (qRT-PCR), statistical analysis	miR-184 was significantly upregulated in GHPAs Highlighted several miRNAs as potential biomarkers	Therapeutic prognostic	[43]
Prostate	-	52 normal tissue samples 499 PC tissue samples Cell lines	Transfection, functional assays, RNA and protein analysis	miR-184 suppressed PC cell proliferation, migration and invasion	Therapeutic	[9]
	-	30 PC and normal tissue samples Cell lines 20 male nude mice	Cell transfection, RT-qPCR, colony formation assay, trans-well assay, dual luciferase reporter assay, RIP assay, western blotting, H And E stain and IHC, in vivo experiments	miR-184 suppresses IGF-1R expression, inhibiting proliferation, migration and invasion	Therapeutic	[18]
	Undetermined	Cell lines	Cell viability, apoptosis, wound healing, migration assays	UCA1 acts as a sponge for miR-184, and ART’s effects are mediated through the miR-184/Bcl-2 signalling pathway	Therapeutic	[20]
	-	Cell lines	Cell viability, colony formation, transwell, wound healing, western blot assays, exosome analysis	MYU upregulates c-MYC by sponging miR-184. overexpression of miR-184 inhibits PC cell proliferation and migration by downregulating c-MYC	Therapeutic	[44]
	-	44 LAPD tissue samples – 24 negative lymph nodes and 20 positives	miRNA sequencing	miR-184 expressed less in patients with lymphatic dissemination. downregulated more with more invasion. suggests possible inhibitory effect on invasion and migration	Prognostic	[45]
Kidney	-	Cell lines	Cell culture, transfection, real-time PCR, MTT assay, scratch assay, apoptosis assay	miR-184 significantly inhibits cell migration and proliferation and promotes apoptosis	Therapeutic	[16]
	-	56 ccRCC and normal tissue samples Cell lines 24 nude mice	Bioinformatics analysis, cell culture, transfection, qRT-PCR, Functional Assays, In Vivo experiments	Silencing LINC01094 or upregulating miR-184 inhibits cell proliferation, migration, and invasion, and promotes apoptosis in ccRCC cells	Therapeutic	[46]
	-	Cell lines	Data collection, differential expression analysis, functional enrichment analysis, miRNA target prediction, validation	miR-184, along with miR-429 and miR-206, downregulates the expression of CCND1, which is involved in cell cycle regulation and cancer progression	Therapeutic	[47]
	+	Cell lines 50 RC tissue samples and adjacent normal tissues	Cell culture, transfection, real-time PCR, MTT assay, transwell assay, western blotting	miR-184 inhibition decreases cell migration and invasion abilities Inhibition of miR-184 increases apoptosis in RC cells, as indicated by higher caspase-3 activity	Therapeutic	[48]
Breast	-	Cell lines	Cell culture, qRT-PCR functional assays, protein analysis	miR-184 overexpression reduced viability, proliferation, and invasion abilities of BC cells under tripterine treatment	Therapeutic	[8]
	-	pubertal mouse mammary gland TNBC cell lines Orthotopic xenografts primary patient samples	MiRNA profiling, miR-184 reactivation, orthotopic xenograft experiments, interaction studies, methylation analysis	Reactivating miR-184 in TNBC cell lines inhibited proliferation and self-renewal in vitro In vivo, miR-184 delayed primary tumour formation and reduced metastatic burden miR-184 regulated genes in the AKT/mTORC1 pathway	Therapeutic	[14]
	-	Cell lines Balb/c Mice	Exosome isolation, cell culture, RNA sequencing, proliferation and migration assays, in vivo experiments, molecular studies	miR-184-3p identified as a tumour suppressor sorted into exosomes by hnRNPA2B1, facilitating tumour cell proliferation and metastasis Overexpressing miR-184-3p and inhibited tumour growth and metastasis	Therapeutic	[49]
	*See ‘brain’ section of table*					[40]
Skin	-	Cell lines: C12C20, A431 NOD/SCID mice	Colony formation assay, β-catenin inhibitor assay, trans-well migration assay, in vivo experiments	miR-184 suppressed cancer development, decreased migration potential, repressed stem cell proliferation and induced differentiation Inhibited neoplastic phenotype	Therapeutic	[7]
	+	3 tissue samples of each HK, SCC and BCC skin lesions	Punch biopsy, laser capture microdissection, miRNA expression profile analysis	miR-184 induced in SCC but not HK and BCC. indicates metastatic and invasive function due to invasive nature of SCC	Therapeutic	[17]
	Undetermined	30 SC and 23 SA tissue samples	Total RNA analysis via RT-PCR	Overexpression of miR-184 3.5 times higher in SC than SA	None	[50]
Lymphoma	-	Cell lines 5 tissue samples of conjunctival MALT and normal adjacent tissue	Apoptosis assay, transwell assay, qRT-PCR, western blotting, luciferase reporter assay	Exogenous miR-184 analogue promoted apoptosis, and inhibited survival, migration, and invasion	Therapeutic	[51]
	-	31 CNSL tissues specimens Cell lines Nude mice	Plasmids construction, lentivirus production and infection, transwell assay, CCK-8 assay, western blotting, immunohistochemical analysis, RNA extraction and RT-PCR assay, luciferase reporter assay, statistical analysis, in vivo studies	Exogenous miR-184 inhibited cell survival, invasion and tumour volumes	Therapeutic	[15]
	Undetermined	Human B cell samples: 29 CD19 + human B cell samples Included naïve, germinal centre, and subepithelial B cells	miRNA profiling, data analysis, network analysis	Nonspecific to miR-184	None	[52]
Pancreas	-	120 pairs of PDAC tissues and matched normal adjacent tissues	Transfection, RNA analysis, apoptosis and cell cycle analysis, protein expression analysis	miR-184 was shown to induce apoptosis and inhibit cell survival and proliferation	Therapeutic prognostic	[11]
	+	Cell lines	Transfection, real-time PCR, MTT assay, cell invasion assay, western blotting	Inhibition of miR-184 reduces cell proliferation and invasion abilities and increases the expression of the pro-apoptotic protein caspase-3, promoting apoptosis in PDAC cells	Therapeutic	[53]
	Undetermined	Tissue samples: 120 PDAC tissues and adjacent	Transfection, RNA analysis, protein expression analysis, apoptosis and cell cycle analysis	miR-184 suppresses cell proliferation and induces apoptosis	Therapeutic prognostic	[54]
Stomach	+	37 human GC tissues and adjacent normal tissues Cell lines BALB/c nude mice (n = 12)	Expression analysis, functional assays, protein analysis	The inhibitory effects of circ_0021087 overexpression on GC cell malignancy were reversed by miR-184 mimic, and the suppressive impact of miR-184 silencing was offset by FOSB knockdown	Therapeutic	[55]
	-	Cell lines Tissue SAMPLES: 33 GC and 7 normal nude mice	Cell transfection and lentiviral infection, qPCR, western blotting, functional assays, In vivo experiments	miR-184 upregulation decreased GC cell growth and proliferation	Therapeutic	[56]
Bone	+	Cell lines Nude mice (n = 24)	Gene manipulation, functional assays, in vivo experiments, molecular analysis	MEG3 downregulates miR-184 and downstream effectors of the Wnt/β-catenin pathway Overexpression of MEG3 leads to reduced proliferation, migration, and increased apoptosis miR-184 mimic reverse tumour suppression by MEG3	Therapeutic	[57]
	+	Cell lines Nude mice (n = 20)	Gene expression analysis, cell proliferation assay, cell invasion assay	miR-184 increased tumour cell proliferation and invasion	Therapeutic	[19]
Leukaemia	Undetermined	Cell lines BALB/c nude mice	Drug resistance induction, qRT-PCR, plasmid construction and transfection, luciferase reporter assay, cell viability and apoptosis assays, in vivo experiments	FENDRR acts as a sponge, reducing the interaction of the RNA-binding protein HuR with MDR1. miR-184 competitively binds to FENDRR with HuR, influencing MDR1 activity	Therapeutic	[58]
	+	Cell lines Peripheral blood samples from 57 acute myeloid leukaemia (AML) patients Fifty athymic BALB/c mice	qRT-PCR, lentiviral vector construction, cell viability assay, western blot, invasion assay, bioinformatics, luciferase assay, animal studies	MEG3 was found to interact with miR-184, leading to decreased miR-184 expression MEG3 overexpression resulted in the downregulation of proliferation-associated proteins (PCNA, BCL-2) and invasion-related proteins (MMP9, VEGF)	Therapeutic	[59]
Gastrointestinal (colorectal)	+	Patient tissue samples	Dynamic array™ integrated fluidic circuit (fluidigm), target prediction and validation	miR-184 was found to be upregulated in CRC tissue samples. this suggests that miR-184 may play a role in the pathophysiology of CRC by potentially targeting and downregulating the tumour suppressor MTUS1	Therapeutic	[60]
Gastrointestinal (oesophageal)	Undetermined	Eighteen pairs of OSCC tissues and oesophagus normal tissues adjacent	Genotyping, RNA analysis, Statistical analysis	Functional TNFAIP2 rs8126 genetic variant is a OSCC susceptibility SNP This SNP could disturb binding of miR-184 with TNFAIP2 mRNA and influence TNFAIP2 regulation	Therapeutic	[61]
Endometrial	-	Cell lines 44 endometrial carcinoma and adjacent tissue samples	Transfection, functional assays, RNA extraction and qPCR, statistical analysis	Low miR-184 expression correlated with poor prognosis and lymph node metastasis in EC patients Overexpression of miR-184 suppressed proliferation and invasion of EC cells	Therapeutic prognostic	[62]
Eye	-	15 human Rb tissues and 3 normal retina tissues Cell lines	Transfection, luciferase assay, qRT-PCR western blotting, immunofluorescence, flow cytometry, cell proliferation and viability assays	Overexpression of miR-184 inhibited proliferation, migration, and invasion of RB cells miR-184 enhanced chemosensitivity of RB cells by inducing apoptosis and G2/M phase arrest	Therapeutic	[63]
Nasopharyngeal	-	Cell lines 25 Nude mice	Cell transfection, qRT-PCR, western blot analysis, transwell invasion and migration assays, wound-healing assay, dual-luciferase reporter assay, immunohistochemistry, in vivo experiments	Overexpression of miR-184 inhibits NPC cell invasion and migration miR-184 overexpression reduces tumour size and metastasis	Therapeutic	[12]
Head and neck	+	Cell lines	Reporter assays, western blot analysis, quantitative RT-PCR, phenotypic assays	miR-21, miR-31, and miR-184 co-target the FIH tumour suppressor during HNSCC pathogenesis	Therapeutic	[21]
Mouth	+	Tumour tissue samples and corresponding normal tissues from 30 OSCC patients Cell lines BALB/c nude mice	Cell transfection, RNA isolation and qRT-PCR, cell proliferation and drug sensitivity assays, dual luciferase reporter assays, western blot and apoptosis assays, in vivo studies	Overexpression of UCA1 promoted cell proliferation and increased resistance to cisplatin UCA1 acted as a sponge for miR-184, suppressing expression Downregulation of miR-184 partially reversed effects of UCA1 knockdown on cell proliferation, CDDP sensitivity, and apoptosis	Therapeutic	[64]

## Results

A total of 123 records were identified from two electronic databases: PubMed (n = 61) and Scopus (n = 62). After applying the publication year filter (2014–2024), 108 records remained, with 15 records excluded for being outside the specified time range. Of the remaining 91 records, 17 were excluded as they were systematic reviews or non-original studies. Screening for duplicates identified 69 unique records after 22 duplicates were removed. These 69 studies were then assessed for relevance and accessibility; 14 records were excluded due to inaccessibility, resulting in 55 studies being included in the final systematic review.

The complete study selection and screening process are summarised in the PRISMA flow diagram (Fig. 2), which outlines the stages of identification, screening, eligibility, and inclusion in accordance with PRISMA 2020 guidelines [24].

## Results by cancer type

### Lung cancer

Eight studies investigated the role of miR-184 in various lung cancer types using a range of experimental models. Overall, most studies demonstrated that miR-184 functions as a tumour suppressor, although one study suggested potential tumour-promoting effects. In small-cell lung cancer (SCLC), miR-184 was shown to suppress tumour progression through the regulation of endothelial PAS domain protein 1 (EPAS1) and β-catenin, leading to decreased metastatic potential [25].

Four studies examined non-small cell lung cancer (NSCLC) and reported consistent findings that miR-184 acts as an anticancer and tumour-suppressive agent. Lin et al*.* [26] investigated miR-184 in conjunction with miR-21, identifying their potential as therapeutic and prognostic biomarkers. Tumour suppression occurred via direct targeting of cell division cycle 25 A (CDC25A) and c-MYC. Similarly, other studies confirmed the tumour-suppressive function of miR-184 both in vitro and in vivo, with c-MYC identified as a recurrent target and potential therapeutic focus [10].

Tung et al*.* [27] explored the influence of miR-184 on cisplatin-based chemotherapy resistance in HPV-associated NSCLC. They found that inhibition of miR-184 increased resistance to cisplatin, indicating that miR-184 promotes chemosensitivity by targeting B-cell lymphoma 2 (Bcl-2). Moreover, next-generation RNA sequencing revealed miR-184 downregulation in squamous cell carcinoma (SCC) and adenocarcinoma (AC), both NSCLC subtypes, compared to normal lung tissue, supporting its tumour-suppressive role [28].

In a related study, Li et al*.* [29] examined idiopathic pulmonary fibrosis (IPF), a condition that elevates lung cancer risk by promoting epithelial–mesenchymal transition (EMT). miR-184 was shown to inhibit transforming growth factor beta 1 (TGF-β1)-induced EMT, thereby reducing metastatic potential and reinforcing its tumour-suppressive activity.

Natasya and Agustriawan [30] assessed the relationship between miRNAs, ethnicity, and cancer stage in lung adenocarcinoma (AC). While the role of miR-184 as a tumour suppressor or promoter was not clearly defined, it was identified as a potential diagnostic and prognostic biomarker.

Conversely, one study reported opposing findings, suggesting that miR-184 may act as a tumour promoter by enhancing tolerance to epidermal growth factor receptor (EGFR)-targeted therapies, thereby facilitating tumour progression when such treatments are inhibited [31].

### Liver cancer

Seven studies investigated the role of miR-184 in liver cancers, particularly hepatocellular carcinoma (HCC). Most studies suggested that miR-184 acts as a tumour promoter, though some presented conflicting evidence indicating possible tumour-suppressive effects.

Guo et al. [32] and Li et al. [33] investigated interactions between miR-184 and circular RNAs, circITCH and circ-102,166, respectively. Both circular RNAs acted as tumour suppressors in HCC by sponging miR-184, which functioned as a tumour promoter. Similarly, the long non-coding RNA (lncRNA) LINC00205 acted as a competitive endogenous RNA (ceRNA), inhibiting miR-184 activity. This interaction enhanced tumour development by upregulating epoxide hydrolase 1 (EPHX1), thereby promoting proliferation, migration, and metastasis in HCC [34]. Further investigation into the effects of miR-184 on HCC revealed that it promotes tumorigenicity via regulation of SRY-box transcription factor 7 (SOX7), influencing the Wnt/β-catenin signalling pathway [22]. Together, these findings indicate a tumour-promoting role for miR-184 in HCC through modulation of oncogenic signalling and ceRNA interactions.

Conversely, Zhang et al. [35] conducted a protein interaction network analysis and found that miR-184 regulates signalling pathways and cellular processes involved in HCC progression, including the cell cycle, apoptosis, and the mechanistic target of rapamycin (mTOR) pathway. Their analysis suggested that miR-184 may act as a tumour suppressor, being downregulated in HCC and associated with oncogenic pathway inhibition.

Two additional studies did not clearly define whether miR-184 functioned as a tumour promoter or suppressor [36, 37]. Computational predictions identified high-confidence interactions between miR-184 and Ras protein-specific guanine nucleotide-releasing factor 1 (RAGRF1), as well as maternally expressed gene 3 (MEG3), the most frequently predicted target [36]. Another study demonstrated that miR-184 was the most upregulated miRNA in exosomes derived from HCC cells treated with tunicamycin, an endoplasmic reticulum (ER) stress inducer, but was not associated with programmed death ligand 1 (PD-L1) expression. Increased expression of ER stress markers was negatively correlated with overall survival and clinicopathological outcomes [37].

Overall, while most studies support a tumour-promoting function of miR-184 in HCC through regulation of oncogenic pathways and ceRNA networks, some evidence suggests potential tumour-suppressive activity under specific molecular contexts.

### Brain cancer

Six studies investigated the role of miR-184 in brain tumours, revealing predominantly tumour-suppressive effects, though one study reported oncogenic activity depending on the molecular context.

Most studies (n = 4) identified miR-184 as a tumour suppressor in glioma. Emdad et al. [38] showed that miR-184 acted as a promising tumour suppressor, while staphylococcal nuclease and tumour domain containing 1 (SND1) functioned as a tumour promoter.

Similarly, Cheng et al. [39] demonstrated that miR-184 overexpression reduced glioma tumour size and induced apoptosis and cell cycle arrest by targeting tumour necrosis factor alpha-induced protein 2 (TNFAIP2). Additional study confirmed miR-184 suppressed tumour development by upregulating pro-apoptotic proteins (caspases-3,8) and tumour suppressor genes (p53, p21) [40].

Furthermore, Liu et al. [41] investigated the prognostic relevance of miR-184 in various astrocytoma subtypes. Low miR-184 expression correlated with advanced tumour grade, older patient age, lower Karnofsky performance score, and higher Ki-67 index. Importantly miR-184 was identified as an independent prognostic biomarker, with reduced expression predicting poorer outcomes.

In contrast, Yuan et al. [42] reported that miR-184 acted as a tumour promoter in glioma, malignant behaviours, including proliferation, invasion, and migration. Mechanistically, miR-184 targeted factor inhibiting hypoxia-inducible factor 1 (FIH-1), which in turn stabilised hypoxia-inducible factor 1-alpha (HIF-1α) and promoted tumour progression. Finally, a study of pituitary adenomas found miR-184 upregulated in growth hormone-secreting pituitary adenoma (GHPA) but did not define a clear functional role in tumourigenesis [43].

Overall, these findings suggest that miR-184 primarily acts as a tumour suppressor in gliomas and astrocytomas, although its role may shift toward oncogenic in specific hypoxia-related or tumour-type-dependent contexts.

### Prostate cancer

miR-184 generally demonstrated tumour-suppressive effects in prostate cancer (PC). Tan et al. [9] reported that overexpression of miR-184 reduced PC cell proliferation, invasion, and metastasis, partly through regulation of deltex-1 (DTX1).

Xie et al. [18] showed that lncRNA small nucleolar RNA host gene 11 (SNHG11) promotes PC progression by sponging miR-184 and upregulating insulin-like growth factor receptor 1 (IGF-1R), whereas miR-184 counteracted these effects; inhibiting proliferation and migration in vitro and in vivo. Similarly, lncRNA MYU promoted malignant growth via the miR-184/c-MYC axis, but overexpression of miR-184 downregulated c-MYC, suppressing tumour growth and metastatic potential [42].

Pudova et al. [45] observed higher miR-184 expression in non-disseminated versus lymphatically disseminated locally advanced PC (LAPC), supporting its tumour-suppressive role.

Zhou et al. [20] investigated the effects of artesunate (ART) on PC through the urothelial carcinoma-associated 1 (UCA1) gene, which functions as a miR-184 sponge. ART treatment reduced tumour growth by inducing apoptosis and inhibiting metastasis. While the study did not define the independent role of miR-184, the findings suggest that the UCA1–miR-184 interaction contributes to tumour suppression during ART treatment. Overall, these studies indicate that miR-184 predominantly acts as a tumour suppressor in PC, regulating key oncogenic pathways and counteracting lncRNA-mediated tumour-promoting mechanisms.

### Kidney cancer

miR-184 predominantly acted as a tumour suppressor in renal cancers, although some studies report context-dependent tumour-promoting effects. Su et al. [16] demonstrated that miR-184 inhibited proliferation and migration while promoting apoptosis in renal cell carcinoma (RCC), supporting its tumour-suppressive role.

Xu et al. [46] investigated LINC01094 in clear cell RCC (ccRCC) and its interaction with miR-184 and SLC2A3. Silencing LINC01094 or upregulating miR-184 reduced proliferation, migration, and invasion, decreased metastatic potential, and promoted apoptosis. miR-184 was also found to be downregulated in ccRCC tissues and directly targeted Cyclin D1 (CCND1), a key regulator of the cell cycle, further confirming its tumour-suppressive function [47]. Conversely, Yang et al. [48] examined multiple renal carcinoma subtypes, including ccRCC, papillary RCC (PRCC), and medullary carcinoma (MC). In this study, miR-184 inhibition reduced cell proliferation and invasion, indicating a potential tumour-promoting role in certain renal cancer contexts. Overall, these findings suggest that while miR-184 acts as a tumour suppressor in ccRCC through regulation of cell cycle and apoptotic pathways, its role may vary depending on the renal carcinoma subtype and molecular context.

### Breast cancer

Three studies evaluated the role of miR-184 in breast cancer and consistently reported tumour-suppressive effects across in vitro, in vivo, and exosomal-transfer models.

Wang [8] demonstrated that miR-184 synergises with the natural compound tripterine to suppress breast cancer cell proliferation, viability, and invasiveness. Mechanistically, tripterine reduced Bcl-2 expression while miR-184 increased Bax, promoting apoptotic pathways. The synergistic interaction between tripterine and miR-184 indicated that miR-184 contributes to BC suppression by reducing tumour cell survival.

Similarly, Phua et al. [14] profiled miRNA expression in mouse mammary gland models and identified miR-184 as a tumour suppressor that reduced metastasis and delayed primary tumour formation. These effects were attributed to modulation of the AKT/mTORC1 signalling pathway, highlighting miR-184’s role in attenuating oncogenic signalling.

In a complementary exosomal study, Zhang et al. [49] demonstrated that tumour cell-derived exosomes enriched with miR-184-3p inhibited tumour growth and metastasis. miR-184-3p was shown to be selectively packaged into exosomes by heterogeneous nuclear ribonucleoprotein A2/B1 (hnRNPA2B1) and to suppress M2 macrophage polarisation, thereby reducing pro-tumour immune responses and metastatic potential.

Collectively, these findings consistently indicate that miR-184 acts as a tumour suppressor in breast cancer, reducing proliferation, enhancing apoptosis, and limiting metastasis through multiple molecular mechanisms and intercellular communication pathways.

### Skin cancer

Three studies explored the role of miR-184 in skin cancers, revealing variable effects depending on cancer subtype and experimental model.

Turovsky et al. [7] investigated miR-184 in squamous cell carcinoma (SCC) using both in vitro and in vivo models. The study demonstrated that miR-184 inhibited cancer cell proliferation, migration, and invasion. In vivo, these effects were confirmed, with miR-184 shown to suppress the neoplastic phenotype of SCC, indicating a tumour-suppressive function.

Conversely, Al-Eryani et al. [17] analysed miRNA expression profiles in three arsenic-induced skin conditions: hyperkeratosis (HK), squamous cell carcinoma (SCC), and basal cell carcinoma (BCC). miR-184 expression was markedly higher in SCC compared to HK and BCC. Given that SCC exhibits greater metastatic potential, the authors suggested that miR-184 may contribute to tumour progression and metastasis, implying an oncogenic role in this context.

Tetzlaff et al. [50] compared total RNA expression profiles between sebaceous carcinoma (SC) and sebaceous adenoma (SA). SC exhibited higher miR-184 expression than SA, although the study did not determine whether miR-184 functioned as a tumour promoter or suppressor. Overall, these findings highlight the context-dependent nature of miR-184 in skin cancers, acting predominantly as a tumour suppressor in SCC models, but potentially exhibiting oncogenic properties under specific pathological or environmental conditions, such as arsenic exposure.

### Lymphoma

Three studies investigated the role of miR-184 in lymphomas, consistently identifying its tumour-suppressive functions across different models and subtypes.

Li et al. [51] examined the involvement of miR-184 in conjunctival mucosa-associated lymphoid tissue (MALT) lymphoma, focusing on its regulation of RasL10B and tumour necrosis factor alpha-induced protein 8 (TNFAIP8). Overexpression of miR-184 inhibited migration, invasion, and cell survival while promoting apoptosis. Conversely, inhibition of miR-184 reversed these effects, confirming its tumour-suppressive role in MALT lymphoma.

Liang et al. [15] explored the function of miR-184 in central nervous system lymphoma (CNSL) and found that exogenous miR-184 reduced tumour invasion, cell survival, and tumour volume in vivo. Mechanistically, miR-184 suppressed the PI3K/AKT signalling pathway by targeting iASPP, thereby enhancing apoptosis and inhibiting proliferation.

In addition, a separate study reported that miR-184 is involved in late B-cell development, with its downregulation observed in marginal zone lymphoma. The loss of miR-184 expression was associated with enhanced tumour development, further supporting its tumour-suppressive role [52].

Collectively, these findings suggest that miR-184 acts as a tumour suppressor in lymphomas, inhibiting proliferation, invasion, and survival through pathways regulating apoptosis and oncogenic signalling. Its consistent downregulation across different lymphoma subtypes highlights its potential as a diagnostic and therapeutic biomarker.

### Pancreatic cancer

Three studies investigated the role of miR-184 in pancreatic ductal adenocarcinoma (PDAC), revealing both tumour-suppressive and tumour-promoting effects depending on the experimental context.

One study demonstrated that miR-184 promoted apoptosis by increasing the expression of caspase-3 and caspase-9 while decreasing Bcl-2, thereby suppressing PDAC cell survival [11]. Inhibition of miR-184 led to increased phosphorylation of PI3K and AKT, promoting proliferation and survival, as well as elevated expression of c-JUN N-terminal kinase 1 (JNK1), contributing to cell growth and apoptotic dysregulation. This indicates that miR-184 can function as a tumour suppressor in PDAC.

Conversely, another study reported a tumour-promoting role for miR-184. Upregulation of miR-184 enhanced PDAC cell proliferation and invasion, while its inhibition reduced these malignant properties and increased apoptosis via elevated caspase-3 activity [53].

Finally, bioinformatics analyses predicted regulatory mechanisms in pancreatic cancer and revealed that miR-184 is downregulated in PDAC tissues compared to normal pancreatic tissues, although no direct functional conclusions were drawn from this study [54]. The effects of miR-184 in PDAC appear context-dependent, with evidence supporting both tumour-suppressive and tumour-promoting roles. Its modulation of apoptotic and proliferation pathways highlights its potential as a therapeutic target, though further mechanistic studies are needed to clarify the conditions under which it exerts each effect.

### Stomach cancer

Two studies investigated the role of miR-184 in gastric cancer (GC), revealing contrasting effects depending on the molecular context. Yu et al. [55] reported that miR-184 was upregulated in GC tissues, whereas Circ_0021087 and FOSB were downregulated. Overexpression of Circ_0021087 suppressed GC cell proliferation, invasion, migration, and epithelial–mesenchymal transition (EMT), indicating that miR-184 may act as an oncogene in this context by promoting tumour progression. Conversely, another study found that lncRNA SNHG11 and CDC25A were upregulated in GC. Silencing SNHG11 increased miR-184 levels while reducing CDC25A expression, leading to decreased cell proliferation, migration, and growth, and inducing apoptosis [56]. These findings indicate that miR-184 functions as a tumour suppressor by targeting oncogenic pathways when SNHG11 is inhibited.

### Bone cancer

MEG3 was reported to downregulate miR-184 in osteosarcoma cells, resulting in reduced proliferation and migration, and increased apoptosis. Tumour-suppressive effects were reversed by a miR-184 mimic, indicating that miR-184 acted as a tumour promoter in this context [57].

Similarly, another study showed that transfection with a miR-184 mimic increased osteosarcoma cell proliferation and metastatic potential compared to cells treated with a miR-184 inhibitor, further confirming the oncogenic role of miR-184 [19].

### Leukaemia

Two studies investigated the role of miR-184 in chronic myeloid leukaemia (CML), focusing on drug resistance and tumour regulation.

Zhang et al. [58] studied miR-184 and human antigen R (HuR) sponging in the context of Adriamycin resistance in vitro and in vivo. miR-184 targeted and decreased MDR1 expression, thereby suppressing drug-resistance mechanisms. These findings also suggested foetal-lethal non-coding developmental regulatory RNA (FENDRR) as a potential target to reverse Adriamycin resistance and enhance therapeutic effectiveness in CML.

Li et al. [59] investigated the tumour suppressor MEG3 and its regulation of miR-184 in CML. MEG3 was downregulated in CML compared to healthy controls and targeted miR-184, reducing its expression. The interaction indicated that MEG3 acts as a tumour suppressor, whereas miR-184 functions as a tumour promoter in this context.

### Other cancers

Seven studies investigated the role of miR-184 in various other cancer types, each focusing on a distinct malignancy and molecular mechanism.

In colorectal carcinoma (CRC), miR-184 was upregulated while microtubule-associated tumour suppressor 1 (MTUS1) was reduced compared to controls. Upregulation of miR-184 contributed to CRC development by downregulating MTUS1, indicating a tumour-promoting role [60].

A study on oesophageal squamous cell carcinoma (oeSCC) examined the TNFAIP2 3′-UTR rs8126 polymorphism, which was associated with increased cancer risk in Chinese populations. The study did not draw conclusions regarding miR-184 function [61]. In endometrial cancer, miR-184 was downregulated compared to normal tissue, with lower expression linked to poorer prognosis and increased metastasis. Overexpression of miR-184 inhibited proliferation and invasion, indicating a tumour-suppressive role [62].

In retinoblastoma (Rb), miR-184 expression was decreased in tumour tissues and chemo-resistant cells. miR-184 acted as a tumour suppressor, reducing metastatic potential, inducing apoptosis and cell cycle arrest, enhancing chemosensitivity by directly targeting SLC7A5, limiting its downstream effects [63].

In nasopharyngeal carcinoma (NPC), miR-184 was downregulated compared to normal epithelium. Overexpression inhibited invasion and migration, suppressing EMT via direct targeting of Notch2, reducing metastatic potential [12].

In head and neck squamous cell carcinoma (hnSCC), miR-184 bound the FIH transcript, reducing FIH protein expression and thereby increasing proliferation and migration, suggesting a tumour-promoting role [21].

Finally, in oral squamous cell carcinoma (orSCC), UCA1 acted as a competitive endogenous RNA (ceRNA), sponging miR-184. Downregulation of miR-184 by UCA1 increased proliferation, cisplatin resistance, and reduced apoptosis, indicating that miR-184 normally functions as a tumour suppressor in this context [64].

**Fig. 3:**
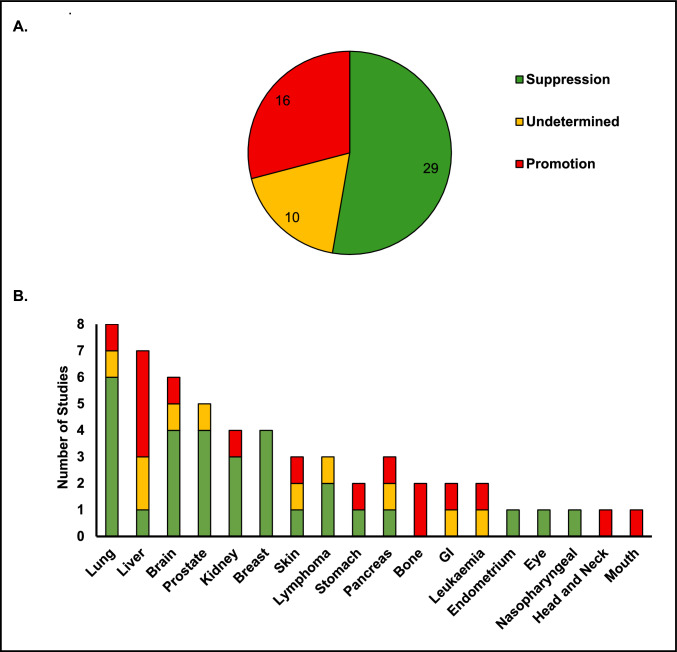
Summary of studies investigating the effects of miR-184 across different cancers. panel (**A**) shows the total number of studies categorised by outcome: tumour suppression, tumour promotion, or undetermined. panel (**B**) presents a stacked column chart displaying the number of studies within each cancer type, grouped by the same three outcomes

**Fig. 4:**
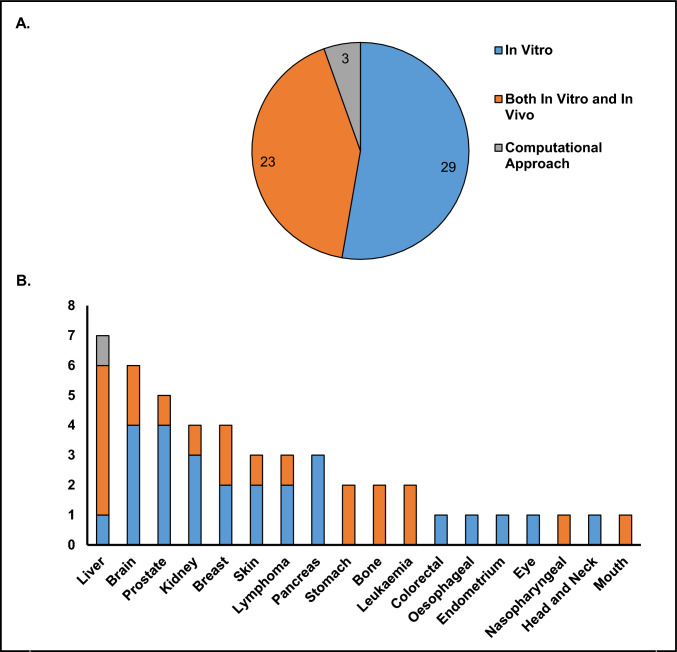
Summary of experimental models used in studies investigating miR-184. panel (**A**) shows the total number of studies for each experimental model, categorised as ‘In Vitro’, ‘both in vitro and in vivo’, or ‘computational approach’. panel (**B**) presents a stacked column chart displaying the number of studies using each experimental model within each cancer type, grouped by the same three classifications

These varied outcomes of the effect of miR-184 on cancer can be seen in Fig. 3 A, B. Summaries of the experimental models used in selected studies on the effect of miR-184 on cancer can be seen in Fig. 4 A, B

A summary of the collated results is presented in Table 1, detailing the characteristics of all included studies, including cancer type, experimental models, investigation methods, main outcomes, and clinical potential.

## Discussion

### Context-dependent role of miR-184 in cancer

This systematic review demonstrates that the role of miR-184 in cancer is highly context-dependent. Across the 55 studies examined, miR-184 exhibits tumour-suppressive, tumour-promoting, or undetermined effects depending on cancer type, experimental model, and cellular environment (Fig. 3 and 4). Its functional outcome is influenced by factors such as signalling pathway interactions, ceRNA networks, and the tumour microenvironment, including exosome-mediated communication. The dual nature of miR-184 underscores the importance of considering tissue-specific and molecular contexts when evaluating its role in cancer.

### Tumour-suppressive roles of miR-184

In several cancers, miR-184 consistently demonstrates tumour-suppressive activity. In prostate cancer, it reduces proliferation, invasion, and migration by targeting DLX1 and IGF-1R, while sponging by oncogenic lncRNAs such as SNHG11 and MYU relieves inhibition of c-MYC, highlighting its suppressive role [9, 18, 44]. Breast cancers show a similar pattern, where miR-184 inhibits proliferation and metastasis, often synergizing with agents like tripterine [8] and maintaining normal mammary epithelial homeostasis [14]. Exosomal transfer further modulates the tumour microenvironment, suggesting a potential mechanism for therapeutic delivery and microenvironmental reprogramming [49]. In kidney cancer (ccRCC), miR-184 suppresses proliferation, migration, and invasion through downregulation of CCND1 and targeting oncogenic pathways, although sponging by LINC01094 and SLC2A3 promotes tumour progression [46, 47]. In pancreatic ductal adenocarcinoma, increased miR-184 promotes apoptosis via caspase-3/9 activation and Bcl-2 downregulation [11], while lymphomas show suppression through inhibition of RasL10B, TNFAIP8, and iASPP, promoting apoptosis [15, 51]. Other cancers, including endometrial cancer, retinoblastoma, nasopharyngeal carcinoma, and oral SCC, also demonstrate tumour-suppressive effects mediated by miR-184 [12, 62–64]. Across these contexts, miR-184 consistently regulates proliferation, apoptosis, migration, invasion, metastasis, and therapy response, functioning as a reliable tumour suppressor and anticancer agent.

### Tumour-promoting roles of miR-184

Conversely, miR-184 can act as a tumour promoter in cancers such as hepatocellular carcinoma (HCC), osteosarcoma, head and neck SCC, and certain contexts of pancreatic cancer. In HCC, miR-184 drives proliferation, migration, and metastasis by regulating SOX7 and Wnt/β-catenin signalling, and ceRNA studies demonstrate that circular RNAs (circITCH, circ-102,166) and lncRNAs like LINC00205 modulate these effects [22, 23, 26–28, 32–34, 65]. Osteosarcoma studies indicate that downregulation of MEG3 enhances miR-184 activity, promoting proliferation and metastasis [19, 57]. In head and neck SCC, miR-184 targets FIH, enhancing proliferation and migration [21]. These findings highlight the oncogenic potential of miR-184 in certain mesenchymal- and hepatocyte-derived cancers, underscoring the necessity of context-specific therapeutic strategies.

The full spectrum of miR-184’s context-dependent activities, synthesising its primary suppressive and promoting pathways, is visually summarised inFig. 5.

**Fig. 5:**
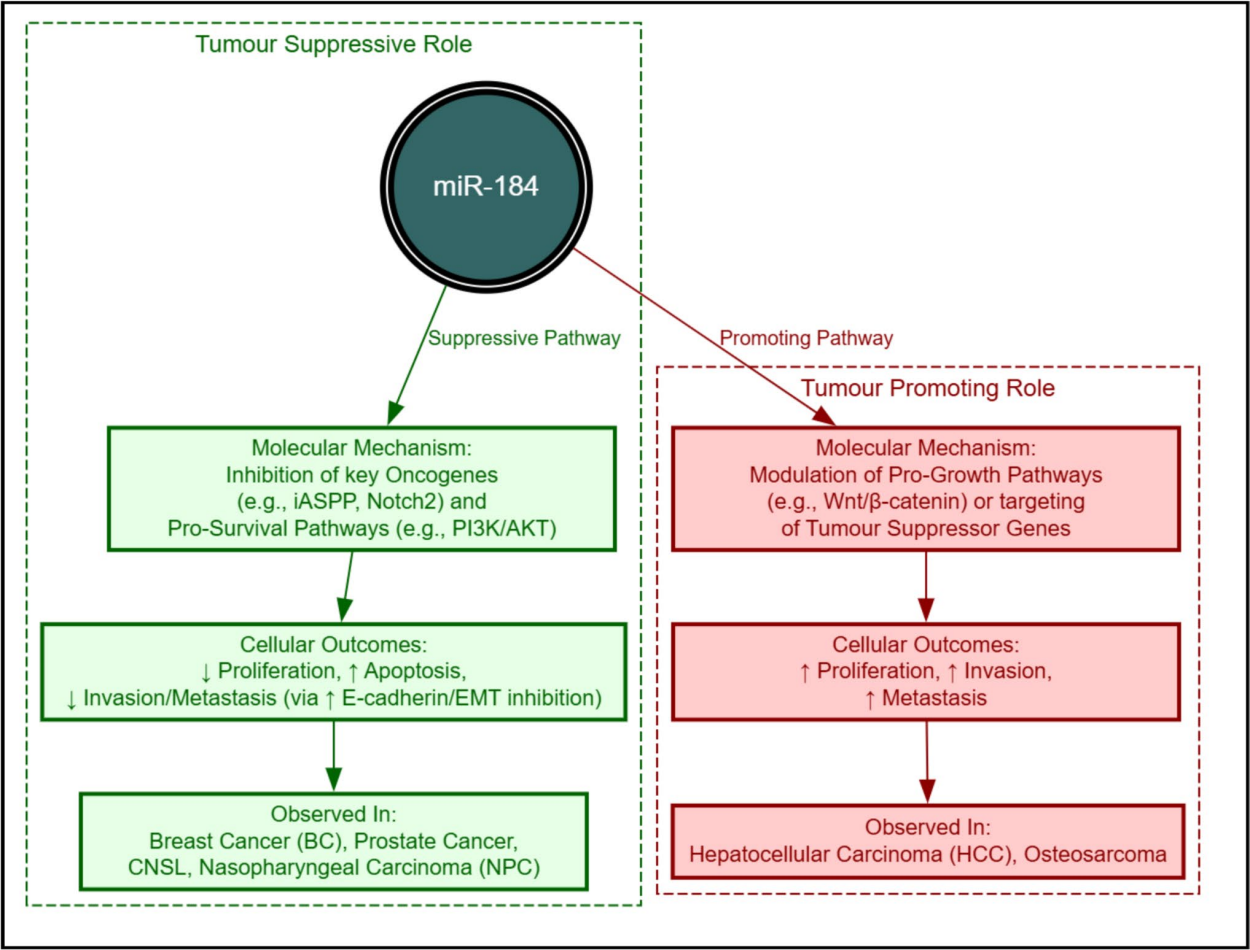
Context-dependent roles of miR-184 in malignancy: suppressive vs. promoting functions. this schematic synthesises the divergent roles of miR-184 identified across cancer types, illustrating the core principle of its context-dependent function.* Left panel—tumour suppressive role:* represents the miR-184 function mediated by pathways such as the inhibition of oncogenes (e.g., iASPP, Notch2) and pro-survival pathways (e.g., PI3K/AKT). cellular outcomes include decreased proliferation and increased apoptosis, commonly observed in cancers such as breast cancer (BC) and prostate cancer (PC). *Right panel—tumour promoting role:* represents the oncogenic function, often mediated by the modulation of pro-growth pathways (e.g., Wnt/β-catenin) or the targeting of tumour suppressor genes. cellular outcomes include increased proliferation, invasion, and metastasis, observed primarily in hepatocellular carcinoma (HCC) and osteosarcoma. The functional outcome of miR-184 is determined by the dominance of these specific regulatory pathways in the tumour microenvironment

### Contradictory or undetermined roles

In some cancers, miR-184 exhibits contradictory or context-dependent effects. Glioma studies show predominantly tumour-suppressive activity via TNFAIP2 and caspase pathways, yet upregulation can enhance proliferation and migration through FIH-1/HIF-1α signalling [39, 42]. Similarly, lung cancers are mostly suppressed by miR-184 via EPAS1, CDC25A, and c-MYC targeting [25, 26], although chemotherapy resistance studies reveal occasional tumour-promoting roles [27, 31]. Renal cancers show split findings, with suppression in ccRCC [16, 47] but promotion in other renal subtypes [48]. n pancreatic and skin cancers, heterogeneity in tumour subtype, microenvironmental factors, and experimental models likely explains observed contradictions [7, 11, 66].

### Mechanistic insights

**Fig. 6:**
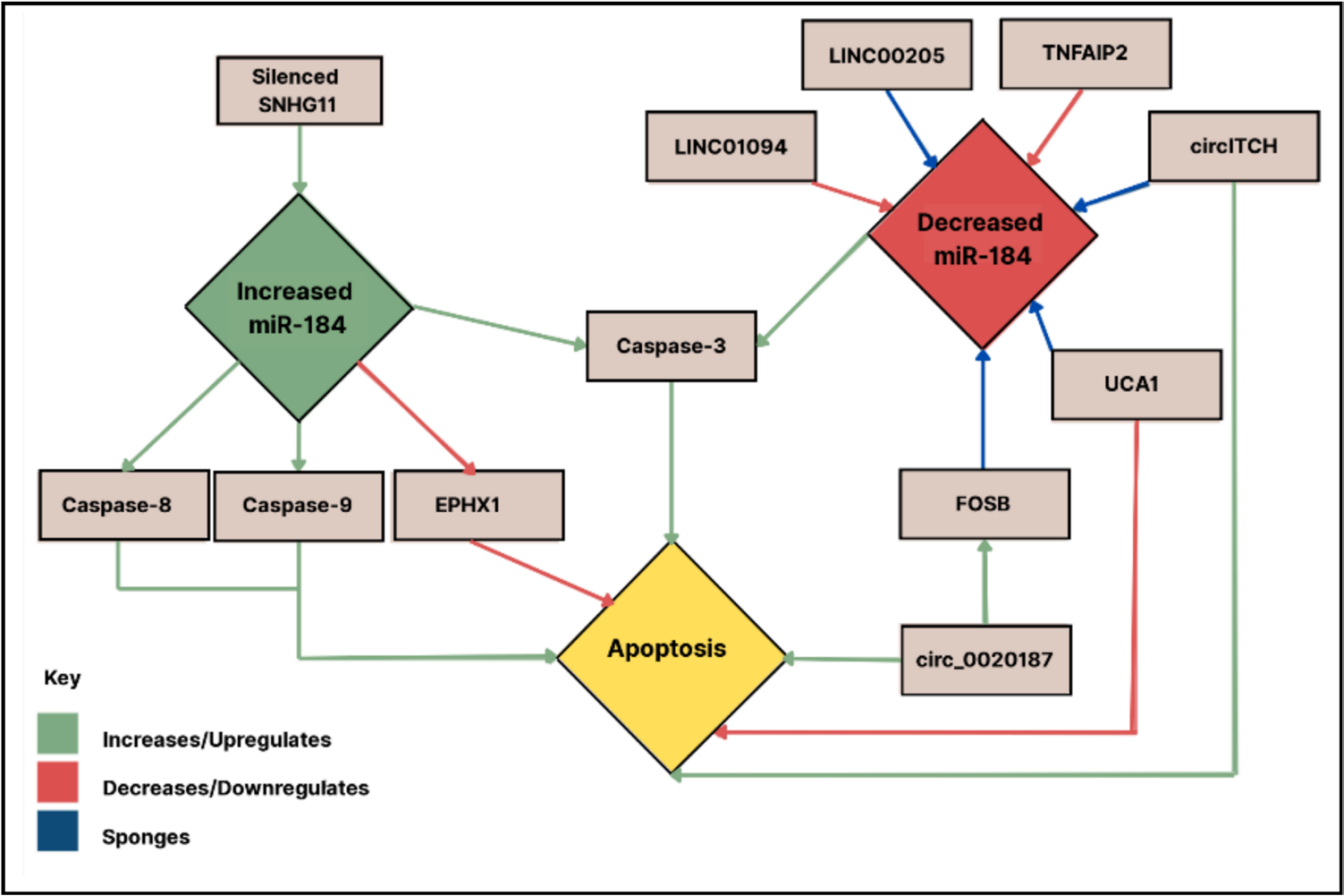
Schematic representation of molecular interactions and pathways involving miR-184 in apoptosis regulation. the diagram illustrates how increased or decreased miR-184 expression influences caspase activation, EPHX1 signalling, and apoptosis, alongside modulation by lncRNAs (LINC00205, LINC01094, TNFAIP2) and circRNAs (circITCH, circ_0020187). Green arrows indicate upregulation, red arrows indicate downregulation, and blue arrows denote sponge interactions

miR-184’s diverse roles across cancer types are mediated through intricate molecular networks that govern cell survival and death. It influences several major signalling pathways, including Wnt/β-catenin, PI3K/AKT, mTOR, and HIF-1α, all of which are key regulators of proliferation, apoptosis, and metastasis [22, 25–27, 42, 47]. Moreover, miR-184 activity is extensively shaped by ceRNA interactions involving lncRNAs (e.g., LINC00205, LINC01094, TNFAIP2) and circRNAs (e.g., circITCH, circ_0020187), which act as molecular sponges that sequester miR-184 and alter its downstream effects [18, 26, 32–34, 44, 46]. The N6-methyladenosine (m6A) modification, for example, has recently been shown to upregulate lncRNA H19, which sponges miR-184 to promote CARM1 expression, thereby driving drug resistance in multiple myeloma [67].These networks converge on apoptotic regulation, where miR-184 has been shown to promote apoptosis via upregulation of caspase-3, caspase-8, and caspase-9 when its expression is increased, while reduced miR-184 expression leads to EPHX1 activation and suppressed apoptosis [49, 53]. Conversely, overexpression of lncRNAs such as LINC00205 or UCA1 can inhibit miR-184, thereby decreasing caspase activity and promoting cell survival. Upstream regulatory mutations in BRCA1 and PALB2 may further influence miR-184’s apoptotic effects in breast and pancreatic cancers [66], illustrating how its function depends on the dominance of specific pathways or ceRNA dynamics within a given tumour context. Figure 6 summarises these molecular interactions, depicting how increased or decreased miR-184 expression differentially regulates apoptotic mediators through both direct targeting and ceRNA sponging relationships.

Taken together, these findings reveal that miR-184 functions within a highly adaptive molecular framework, in which subtle changes in its regulation can determine whether it acts as a tumour suppressor or promoter. This context-dependent plasticity provides a valuable foundation for translational applications, as discussed in the following section on *Therapeutic and Prognostic Implications*.

### Therapeutic and prognostic implications

The dual nature of miR-184 makes it a promising but complex therapeutic target. In cancers where it acts as a tumour suppressor, such as breast and prostate cancers, miR-184 mimics or combination therapies (e.g., tripterine in BC, ART in PC) can enhance treatment efficacy [8, 19]. Exosomal delivery represents an additional strategy to modulate the tumour microenvironment and restore tumour-suppressive effects [49, 53]. In haematological malignancies, miR-184 contributes to chemoresistance, suggesting that targeted inhibition may restore sensitivity to agents like Adriamycin or cisplatin [48, 58]. This includes specific resistance to the proteasome inhibitor bortezomib in multiple myeloma, which is driven by an N6-methyladenosine (m6A)-mediated upregulation of lncRNA H19, functioning as a sponge to regulate the miR-184/CARM1 axis [67]. Circulating miR-184 also shows promise as a biomarker; however, prognostic interpretations are cancer-specific, necessitating careful consideration in clinical contexts.

### Methodological considerations and evidence quality

Most studies employed robust experimental approaches, including RNA and protein analyses, functional assays, and in vivo xenograft models. However, reliance on cell lines introduces limitations, such as genetic drift and incomplete representation of the tumour microenvironment [68]. Tissue samples offer physiological relevance but can be affected by variability in collection and handling [69]. Xenografts better capture microenvironmental influences but carry species-specific differences [70]. No clinical trial data were available, highlighting the need for translational studies [71, 72]. Methodological variability, including the use of single cell lines, limited cohort sizes, and divergent endpoints, may account for contradictory findings in some cancers.

## Final insights and conclusions

This review clarifies that miR-184 does not act as a universal anti-cancer agent. Its role is highly context-dependent, governed by tumour type, cellular environment, molecular signalling, and ceRNA networks. Consistent tumour-suppressive activity is observed in prostate, breast, kidney, and certain haematological cancers, while liver, bone, and some pancreatic cancers show tumour-promoting effects. Contradictory roles in skin, pancreatic, and brain cancers reflect heterogeneity in tumour subtype, experimental models, and microenvironmental influences.

Future research should focus on comprehensive functional studies spanning cell lines to animal models and, where appropriate, clinical trials, particularly for cancers where miR-184 consistently demonstrates tumour-suppressive effects. A deeper understanding of miR-184’s context-dependent mechanisms will be crucial for developing miRNA-based therapeutic strategies and reliable biomarkers, ultimately aiming to improve patient outcomes across cancer types.

## Data Availability

No datasets were generated or analysed during the current study.

## Abbreviations

AC: Adenocarcinoma
ART: Artesunate
BC: Breast cancer
BCC: Basal cell carcinoma
Bcl-2: B cell lymphoma 2
CCND1: Cyclin D1
CDC25A: Cell division cycle 25A
ceRNA: Competitive endogenous RNA
CML: Chronic myeloid leukaemia
CNSL: Central nervous system lymphoma
CRC: Colorectal carcinoma
DTX1: Deltex-1
E-cadherin: Epithelial cadherin
EGFR: Epidermal growth factor receptor
EMT: Endothelial-to-mesenchymal transition
EPAS1: Endothelial PAS domain protein 1
FENDRR: Foetal-lethal non-coding developmental regulatory RNA
FIH: Factor-inhibiting hypoxia-inducible factor
FIH-1: Factor inhibiting hypoxia-inducible factor 1
GC: Gastric cancer
GHPA: Growth hormone-secreting pituitary adenoma
HCC: Hepatocellular carcinoma
HIF-1α: Hypoxia-inducible factor 1 alpha; HK, hyperkeratosis
hnRNPA2B1: Heterogeneous nuclear ribonucleoprotein A2/B1
hnSCC: Head and neck squamous cell carcinoma
HuR: Human antigen R
HPV: Human papilloma virus
IGF-1R: Insulin-like growth factor receptor 1
iASPP: Inhibitory member of apoptosis-stimulating protein of p53
IPF: Idiopathic pulmonary fibrosis
JNK1: C-JUN N-terminal kinase 1
LAPC: Locally advanced prostate cancer
lncRNA: Long non-coding RNA
MALT: Mucosa-associated lymphoid tissue lymphoma
MC: Medullary carcinoma
MEG3: Maternally expressed gene 3
miRNA: MicroRNA
miR-21: MicroRNA-21
miR-184: MicroRNA-184
mRNA: Messenger RNA
mTOR: Mechanistic target of rapamycin
mTORC1: Mechanistic target of rapamycin complex 1
MTUS1: Microtubule-associated tumour suppressor 1
N-cadherin: Neural cadherin
Notch2: Notch receptor 2
NPC: Nasopharyngeal carcinoma
NSCLC: Non-small cell lung cancer
oeSCC: Oesophageal squamous cell carcinoma
orSCC: Oral squamous cell carcinoma
PC: Prostate cancer
PDAC: Pancreatic ductal adenocarcinoma
PD-L1: Programmed death-ligand 1
PI3K: Phosphatidylinositol 3-kinase
PRCC: Papillary renal cell carcinoma
RAGRF1: Ras protein-specific guanine nucleotide-releasing factor 1
Rb: Retinoblastoma
RCC: Renal cell carcinoma
RC: Renal carcinoma
SCC: Squamous cell carcinoma
SCLC: Small cell lung cancer
SC: Sebaceous carcinoma
SA: Sebaceous adenoma
SNHG11: Small nucleolar RNA host gene 11
SND1: Staphylococcal nuclease and tumour domain-containing 1
SOX7: SRY-box transcription factor 2
TGF-β1: Transforming growth factor beta 1
TNFAIP2: Tumour necrosis factor alpha-induced protein 2
TNFAIP8: Tumour necrosis factor alpha-induced protein 8
TNBC: Triple-negative breast cancer
UCA1: Urothelial carcinoma-associated 1
Wnt: Wingless-related integration site
